# HIV fragments detected in Kaposi sarcoma tumor cells in HIV-infected patients

**DOI:** 10.1097/MD.0000000000031310

**Published:** 2022-10-28

**Authors:** Tung-Ying Chen, Horng-Woei Yang, Dar-Shong Lin, Zo-Darr Huang, Lung Chang

**Affiliations:** a Department of Pathology, MacKay Memorial Hospital, Taipei, Taiwan; b Department of Medicine, MacKay Medical College, New Taipei City, Taiwan; c Department of Medical Research, MacKay Memorial Hospital, New Taipei City, Taiwan; d Department of Pediatric, MacKay Memorial Hospital, Taipei, Taiwan; e Division of Genetics, MacKay Children’s Hospital, Taipei, Taiwan; f Division of Infectious Disease, MacKay Children’s Hospital, Taipei, Taiwan.

**Keywords:** HIV, human herpesvirus-8, Kaposi sarcoma, Kaposi sarcoma-associated herpesvirus

## Abstract

Kaposi sarcoma (KS) is a malignant vascular neoplasm caused by KS-associated herpesvirus (KSHV) infection. HIV plays a major role in KS pathogenesis. KS in HIV usually produces more malignant features than classic KS. Despite the close KS–HIV relationship, no study has reported the existence of HIV in KS tissue. We used ddPCR to detect HIV and KSHV in HIV^+^ KS samples and classic KS control. We verified KS cell types through immunohistochemistry and applied hypersensitive in situ hybridization (ISH) to detect HIV and KSHV in tumor cells. Furthermore, we co-stained samples with ISH and immunohistochemistry to identify HIV and KSHV in specific cell types. Regarding pathological stages, the KS were nodular (58.3%), plaque (33.3%), and patch (8.3%) tumors. Moreover, ddPCR revealed HIV in 58.3% of the KS samples. ISH revealed positive Pol/Gag mRNA signals in CD34 ^+^ tumor cells from HIV ^+^ patients (95.8%). HIV signals were absent in macrophages and other inflammatory cells. Most HIV ^+^ KS cells showed scattered reactive particles of HIV and KSHV. We demonstrated that HIV could infect CD34 ^+^ tumor cells and coexist with KSHV in KS, constituting a novel finding. We hypothesized that the direct KSHV–HIV interaction at the cellular level contributes to KS oncogenesis.

## 1. Introduction

Kaposi sarcoma (KS) is a vascular neoplasm first reported by Dr Moritz Kaposi in 1872. It can be clinically classified as classic, African endemic, iatrogenic, and acquired immune deficiency syndrome (AIDS)-associated KS.^[1,2]^ Classic KS mainly occurs in elderly patients, lesions are mostly confined to the skin, although they can also involve mucosa, lymph nodes, and visceral organs.^[3–6]^ In the early 1980s, the incidence of KS increased rapidly with the number of AIDS cases; accordingly, evidence reveals that KS was induced by human immunodeficiency virus (HIV). Notably, KS concurrent with HIV mostly occurs in HIV-infected men who have sex with men (MSM); it rarely occurs in intravenous drug users or blood donors.^[7]^ KS was suspected to be caused by another sexually transmitted virus. Chang et al^[8]^ identified a new type of human herpesvirus (HHV-8), which is also termed KS-associated herpesvirus (KSHV). Studies have reported that nearly all patients with KS tested positive for the latency-associated nuclear antigen (LANA) of KSHV through immunohistochemistry (IHC), indicating that KSHV is latent in KS cells.^[9,10]^ Therefore, the presence of KSHV is a diagnostic requirement for KS in clinical practice.^[11]^

KS tumor cells are spindle shaped and exhibit characteristics of endothelial and mesenchymal cells. These characteristics are believed to be related to the endothelial-to-mesenchymal transition induced by KSHV.^[12]^ Moreover, KSHV encodes multiple oncogenes such as viral G protein-coupled receptor, which induces DNA damage, viral interferon regulatory factors, which serve the function of immune evasion, and expresses mutated tumor suppressor gene p53.^[13,14]^ KS is considered the most prevalent malignancy in patients with AIDS. Although the close relationship between HIV and KSHV in terms of KS induction has been identified for decades, a detailed explanation of the carcinogenesis of KS has yet to be investigated.

HIV acts as a cofactor in the carcinogenesis of KS.^[15]^ Individual genetic susceptibility and immune status also play a critical role in the carcinogenesis of KS.^[16]^ However, the literature does not contain reports documenting HIV presence in KS tumor tissue.^[17]^ Accordingly, we conducted this study to explore the presence of HIV in KS by using refinement molecular and IHC methods.

## 2. Materials and Methods

### 2.1. Patients and samples

We included patients who were diagnosed as having KS in the skin or visceral organs (ICD-10CM: C46, 46.*) during 2000-2020. We requested formalin-fixed, paraffin-embedded (FFPE) section samples collected from these patients. All samples were confirmed through LANA to be KSHV-KS. Samples that were HIV ^+^ were assigned to the experimental group, and those that were HIV^−^ were assigned to the control group. Table 1 lists the demographic data of all patients. Our study was approved by the Institutional Review Board of MacKay memorial hospital (21MMHIS302e).

**Table 1:** Demographic characteristics of patients with KS.

Case no.	Gender	Age	DoC	HIV serology	KSHV LANA	HIV load	Tissue	Pathology stage
1	M	34	2010/2	+	+	High	Skin	Patch
2	M	33	2011/3	+	+	Moderate	Skin	Nodular
3	M	50	2017/4	+	+	High	Skin	Nodular
4	M	35	2017/2	+	+	Unknown	Skin	Nodular
5	M	47	2016/9	+	+	Low	Lung	Nodular
6	M	33	2016/9	+	+	High	Skin	Plaque
7	M	34	2014/12	+	+	Unknown	Skin	Plaque
8	M	34	2014/12	+	+	Moderate	Skin	Nodular
9	M	45	2014/9	+	+	High	Skin	Nodular
10	M	32	2014/6	+	+	High	Skin	Patch
11	M	38	2013/11	+	+	Low	Skin	Plaque
12	M	44	2012/6	+	+	High	Skin	Nodular
13	M	25	2010/3	+	+	Low	Skin	Plaque
14	F	6	2006/6	+	+	Low	Spleen	Nodular
15	M	28	2006/1	+	+	Unknown	Skin	Nodular
16	M	46	2005/1	+	+	Low	Skin	Plaque
17	M	54	2003/8	+	+	Unknown	Skin	Nodular
18	M	29	2018/1	+	+	Low	Skin	Plaque
19	M	34	2013/4	+	+	Low	Skin	Nodular
20	M	28	2015/3	+	+	Low	Skin	Nodular
21	M	30	2018/6	+	+	Low	Skin	Nodular
22	M	35	2016/5	+	+	Low	Skin	Plaque
23	M	26	2018/6	+	+	High	Skin	Plaque
24	M	50	2019/1	+	+	High	Skin	Plaque
C1	M	70	2017/6	-	+	Nil	Skin	Nodular
C2	F	82	2017/3	-	+	Nil	Skin	Nodular
C3	M	66	2017/2	-	+	Nil	Skin	Nodular

Viral load of >100,000 = high; 1000 < viral load < 100,000 = moderate; and <1000 copies = low.

DoC = date of collection, F = female, KS = Kaposi sarcoma, KSHV = Kaposi sarcoma–associated herpesvirus, LANA = latency-associated nuclear antigen, M = male.

### 2.2. DNA extraction from FFPE section samples

We used a QIAamp DNA FFPE Tissue Kit (QIAgen GmbH, Hilden, Germany) to extract total DNA in accordance with the manufacturer’s instructions. We measured DNA quality and concentration by using a Nanodrop 2000 Spectrophotometer (Thermo Fisher, Waltham, MA).

### 2.3. Virus fragment detection using droplet digital polymerase chain reaction

We used a droplet digital polymerase chain reaction (ddPCR) system (QX200, Bio-rad, Hercules, CA) to detect viral fragments. For this detection, 10 µL of 2× ddPCR Supermix for Probes (no dUTP), 0.15 µL of 100 µM target-specific primer pairs, 0.05 µL of target-specific probe sets were used; Pol (FAM, blue) and Gag (HEX, green)^[18]^ luminous probes (Table S2, Supplemental Digital Content 1, http://links.lww.com/MD/H759) were also used. Moreover, we used at least 20 µg of purified FFPE DNA samples. Finally, we added 20 µL of distilled water to each reaction well. A positive control sample of Pol and Gag (pLP1, 1 × 10^–10^ μg/mL) was provided by Dr Chang (Institute of Microbiology and Immunology, Yang Ming Chiao Tung University, Taiwan). The PCR program was as follows: 95°C for 10 minutes, 94°C for 30 seconds, 55°C for 1 minute (39 cycles, ramp 2 °C/s), and 98°C for 10 minutes, followed by maintaining the reaction at 12°C. The fluorescence intensity level in each well was analyzed using QuantaSoft analysis software (v. 1.7; Bio-rad, Hercules, CA). The droplets were classified as positive or negative according to fluorescence amplitudes. We determined the target concentration by adjusting fluorescence intensity by using a Poisson algorithm according to the manufacturer’s instructions.^[19]^

### 2.4. Blood HIV-1 quantification test

Blood sample of HIV quantification test was performed by cobas 4800 HIV-1 system (Roche Molecular system). We defined blood HIV viral load from high to low according to real-time PCR results: high viral load if PCR copies number more than 100,000 copies/mL, moderate for PCR copies between 1000 and 100,000 copies/mL, low viral load for PCR copies less than 1000 copies/mL.^[20,21]^

### 2.5. In situ hybridization

For in situ hybridization (ISH), we used a RNAscope 2.5 duplex detection kit and chromogenic probes (Advanced Cell Diagnostics, Hayward, CA) to detect HIV and KSHV signals in the KS samples. The detection process was conducted in accordance with the manufacturer’s instructions. HIV and KSHV reactive particles were marked in red and green. Images were captured using an ECLIPSE Ni-U microscope and processed using NIS-Elements D software (Nikon, Tokyo, Japan).

### 2.6. Immunohistochemistry process

The IHC process was performed in accordance with the EnVision + Dual Link System-HRP (DAB+) protocol (Dako, Santa Clara, CA). After the sections were hybridized with C1 and C2 probes, they were washed with buffer, immersed in a dual endogenous enzyme block for 5 minutes, washed with distilled water, incubated with the primary antibodies anti-CD4 (1:100), anti-CD8 (1:100), anti-CD19 (1:100), anti-CD34 (1:100), and anti-CD68 (1:100) for 30 minutes at room temperature; and then washed with distilled water 3 times.^[22]^ They were subsequently incubated with secondary antibodies conjugated to polymer-HRP reagent for 30 minutes and then washed with buffer solution for 5 minutes. The sections were processed through a cascade of C1 and C2 signal amplification procedures and then subjected to a colorimetric reaction with 3,3′-diaminobenzzidine for detecting CD4, CD8, CD19, CD34, and CD68 expression; subsequently, the sections were counterstained with hematoxylin. Finally, they were imaged as aforementioned.

### 2.7. Statistical analysis

To measure the number of positive signals among the KS samples with IHC and ISH methods, we recorded the findings under high-power field of light microscope (HPF, 400×). Directly counting positive signals were added up after manually evaluated under ten different HPF by a pathologist (TYC).

## 3. Results

### 3.1. Demographic characteristics of patients with KS

Total 27 samples were included in this study which contained 24 patients of KS with HIV-infected (HIV^+^) and 3 patients of classic KS (HIV^-^) as control group (Table 1). The age of control group was elder (range 66–82 years), pathologic stage identified as nodular type. Only one patient in the HIV ^+^ group was a female; the rest were males. The average age of the patients was 35.4 years (range 6–54 years). Most of the KS samples (25/27, 92.6%) were obtained from the skin, except for one from the lung and one from the spleen. Regarding the pathological stages of the KS, 58.3% of the tumors were nodular, 33.3% were plaque, and 8.3% were patch stage. HIV viral load data were available for 83.3% of the patients, 50% of the patients had a low viral load due to antiretroviral therapy (ART). No significant interrelation between tumor stage and HIV viral load was found in this study.

### 3.2. HIV viral DNA detected in KS patient

**Figure 1.**
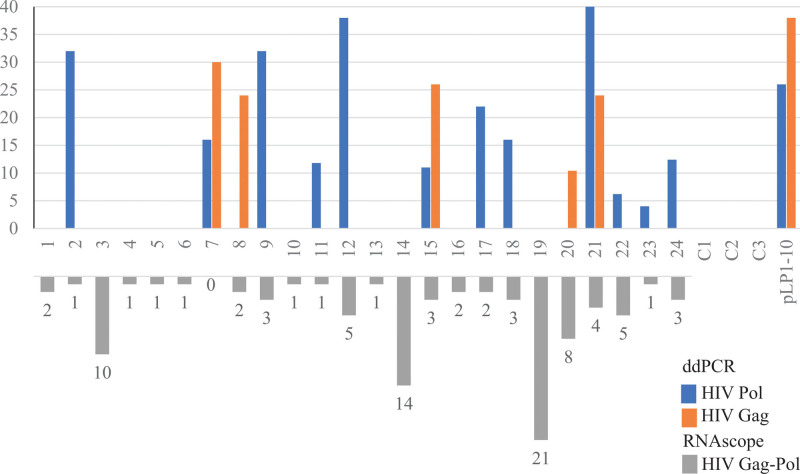
HIV detection results in ddPCR and RNAscope. Samples 1 to 24 represent HIV ^+^ KSHV-KS. C1 to C3 represent the HIV^−^ KSHV-KS control. pLP1^-10^ represents a plasmid control containing HIV Pol and Gag fragments. The *Y*-axis indicates the ddPCR results (copies/20 µL/well). The blue bar represents HIV Pol, and the orange bar represents HIV Gag. The gray bars represent HIV stained with RNAscope and counted using a microscope under a high-power field. Table S1, Supplemental Digital Content 2, http://links.lww.com/MD/H758 lists the score of each sample. ddPCR = droplet digital polymerase chain reaction, KSHV = Kaposi sarcoma-associated herpesvirus.

We detected DNA fragments incorporated or embedded in both HIV ^+^ and HIV^-^ KS samples. All the samples detected had Pol (blue), Gag (orange), or both fragments (Fig. 1). Furthermore, we used ISH to detect HIV DNA fragments in tissue sections directly. The gray bars in Figure 1 demonstrate the results of ISH conducted under a HPF, indicating that HIV viral DNA (vDNA) could be detected in KS, which is a novel finding of our study. The detailed counts of HIV vDNA in all samples are listed in Table S1, Supplemental Digital Content 2, http://links.lww.com/MD/H758. The counts of HIV vDNA in KS were not correlated with HIV viral loads.

### 3.3. Coexpression vDNA and cell markers in KS patient

**Figure 2.**
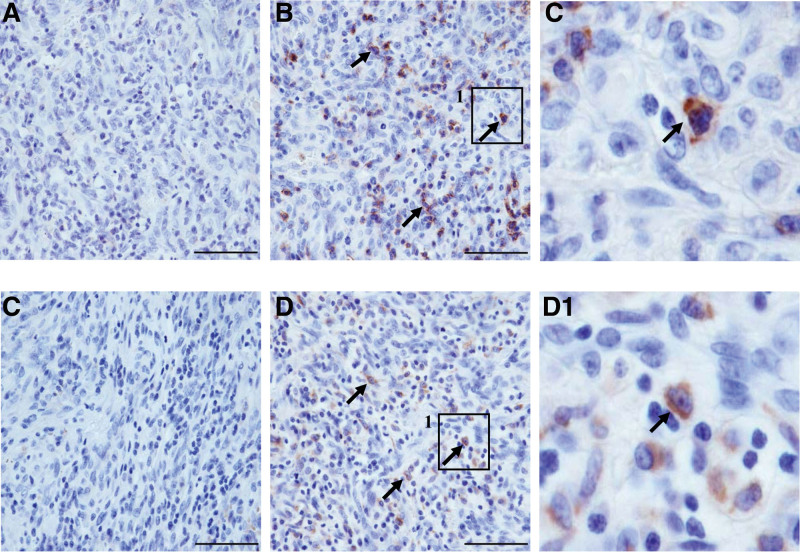
Inflammatory cells in HIV ^+^ KS. Case 15 was used to demonstrate inflammatory cell markers in KS, CD4 (A), CD8 (B, arrow), CD19 (C), and CD68 (D, arrow). B1 and D1 are enlarged versions of the images in the inset boxes in B and D (scale bar = 50 µm). KS = Kaposi sarcoma.

**Figure 3.**
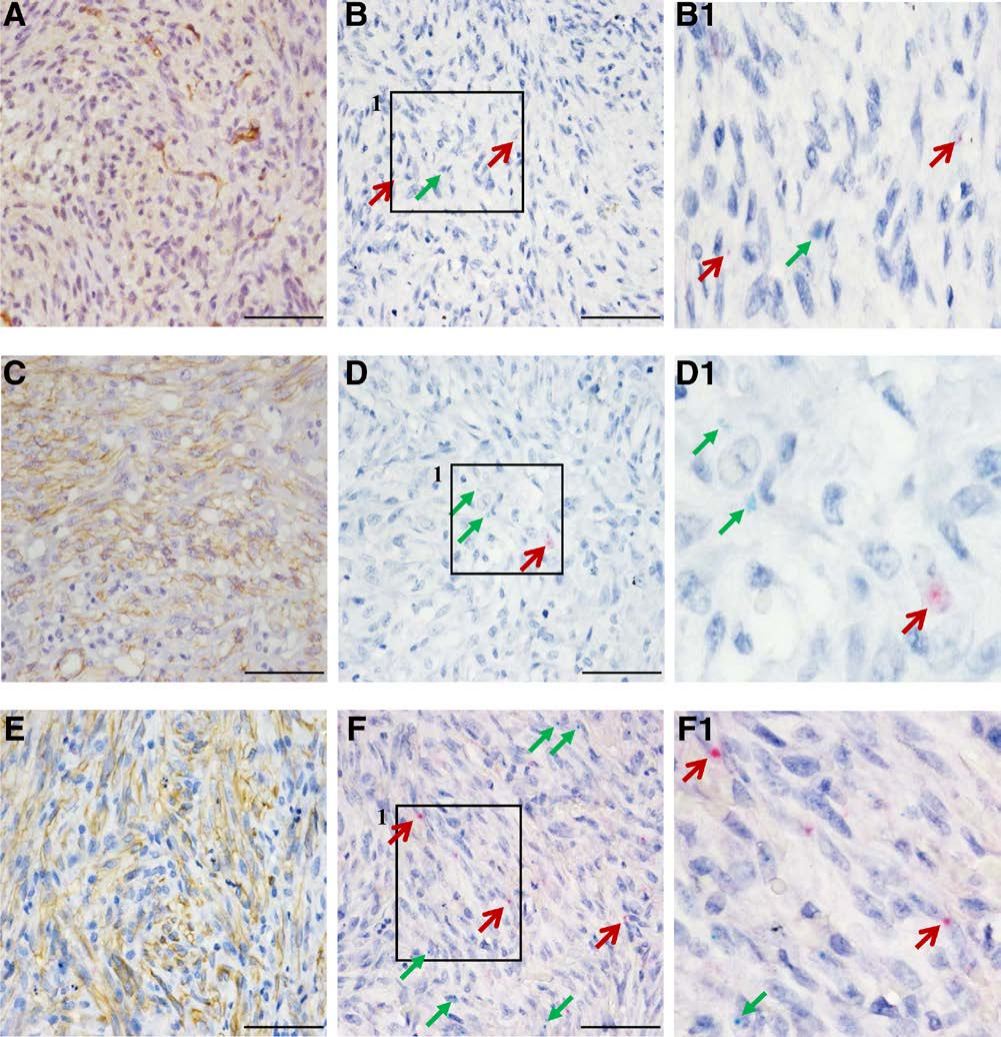
CD34, HIV, and KSHV expression in KS. Fascicles of KS spindle tumor cells are diffusely positive for CD34 (A, C, E; brown). RNAscope shows scattered HIV signals (B, D, F; red arrow) and KSHV signals (B, D, F; green arrow) in tumor cells in cases 14, 15, and 19, respectively. B1, D1, and F1 are enlarged versions of the images in the inset boxes in B, D, and F. Cases 14, 15, and 19 were used as demonstration samples. (scale bar = 50 µm). KSHV = Kaposi sarcoma-associated herpesvirus.

**Figure 4.**
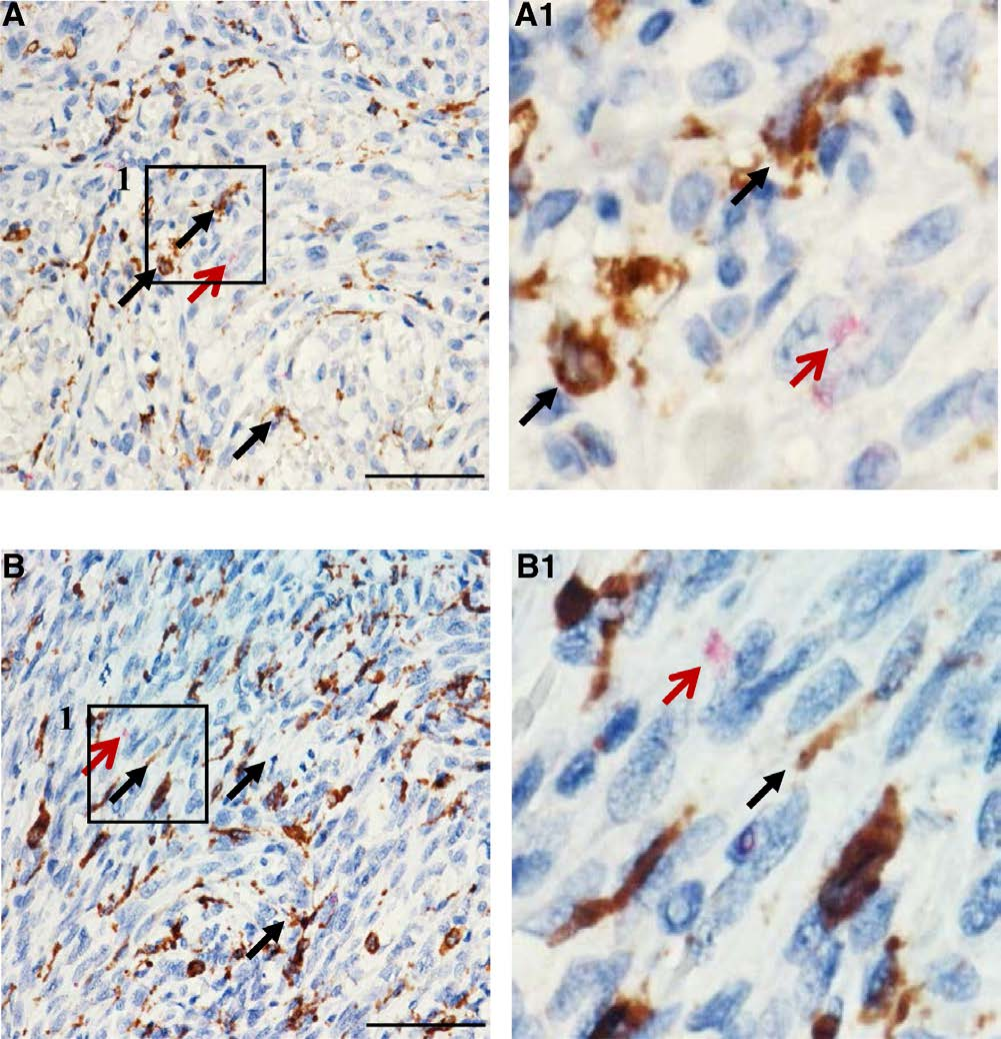
Coexpressed CD68 and HIV in KS. One-step detection of HIV (red arrow) and CD68 (black arrow). In cases 8 (A) and 9 (B), A1 and B1 are enlarged versions of the images in the inset boxes in A and B (scale bar = 50 µm). KS = Kaposi sarcoma.

The number of inflammatory cells infiltrating the tumors varied. We identified the inflammatory cells based on CD4, CD8, CD19, and CD68, which are markers of T helper cell, cytotoxic T-cell, B-cell, and macrophages, respectively. The staining results revealed that T helper cells and B cells were undetected, in contrast to cytotoxic T cells and macrophages, which were prominent (Fig. 2). We confirmed the KS cells to be typical plump spindle tumor cells based on the CD34, which is an endothelial marker (Fig. 3). We found scattered reactive particles of both HIV (red) and KSHV (green) in the tumor cells of most HIV ^+^ KS samples (23/24, 95.8%, Fig. 3, Table S1, Supplemental Digital Content 2, http://links.lww.com/MD/H758) according to cell morphology along with positive CD34 staining. Additionally, HIV signals were absent in lymphocytes and CD68 ^+^ macrophages evidenced by IHC and cell morphology (Fig. 4).

## 4. Discussion

The obscure “relationship” between the oncogenesis of KSHV, HIV in KS has long been an object of research. The most widely accepted theory is that HIV infection leads to insufficient CD4 blood cell counts and the decline of immune function, which engenders KSHV reactivation with the occurrence of KS.^[23]^ However, some researchers have pointed out that the transactivating regulatory protein (Tat) of HIV can cause chronic inflammation and promote KSHV from the latent to the lytic phase and that KSHV angiogenesis reactivates the conversion of epidermal cells into KS cells.^[24]^ In the current study, we used high-sensitivity tools to detect HIV DNA fragments (Pol and Gag) in KS tissue sections obtained from all patients with HIV. Furthermore, we detected HIV ^+^ CD34 ^+^ tumor cells. According to our review of the literature, our study is the first to report this finding.

KS tissue contains several cell types, including typical spindle tumor cells, vascular endothelial cells, and infiltrating inflammatory cells. As expected, the LANA of KSHV was detected in spindle tumor cells positive for CD34, a marker of endothelial cells.^[25]^ Despite CD4 being the primary cell surface receptors for HIV entry into cells, CD4 ^+^ T cells were rarely found in KS. Most of the lymphocytes in KS were CD8 ^+^ T cells, which can be attributed to the antitumor immunity of CD8 ^+^ T cells.^[26]^ Using high-resolution ISH-labeling methods to detect low-abundance transcripts of HIV Pol and Gag fragments, we detected all the positive signal of HIV in CD34 ^+^ spindle cells. Bordoni et al^[23]^ reported that bone marrow CD34 ^+^ progenitor cells might harbor HIV DNA. They indicated that HIV DNA could be detected in CD34 ^+^ hematopoietic progenitor cells in bone marrow, even after a period of ART. Most of the KS with patients in the present study received ART but were still positive for HIV. This may indicate latent phenomenon of HIV in the reservoir cells. However, previous studies have reported that ART reduced the incidence of KS in patients with HIV.^[24,27]^

We marked macrophages through CD68 immunostaining because CD68 ^+^ cells such as macrophages can be infected by HIV.^[28]^ In this study, we observed a prominent quantity of CD68 ^+^ macrophages in the KS samples but did not identify HIV in the CD68 ^+^ cells. Our findings that both KSHV and HIV ^+^ CD34 ^+^ tumor cells prompted 2 further conjectures: HIV in KS directly promotes the carcinogenesis of KSHV or KSHV facilitates the mechanism of HIV entry into CD34 ^+^ tumor cells.

This study has some limitations. First, most patients in the study group were young MSM; this might affect KS disease presentations. Second, we had no KSHV serology data. Furthermore, the ISH and ddPCR results had some discrepancies, which may be due to the diverse FFPE sections and the sensitivity limitations of the detection tools. In addition to the positive ddPCR findings, we used ISH to confirm our hypothesis and delineated the cells infected by HIV. We did not compare the clinical characteristics, histological, and immunological abnormalities between HIV fragment positive patients and negative patients due to small sample size of control group.

In conclusion, this study demonstrated that HIV ^+^ KS tumor cells. These findings suggest that possibility of HIV along with KSHV is directly involved in the promotion of tumor growth at the intracellular level. Further research is required to delineate the molecular mechanism of KS oncogenesis to develop optimal therapeutic options for HIV ^+^ KS patients.

## Author contributions

**Conceptualization:** Tung-Ying Chen, Horng-Woei Yang, and Lung Chang.

**Data curation:** Tung-Ying Chen, Horng-Woei Yang, and Zo-Darr Huang.

**Formal analysis:** Tung-Ying Chen and Horng-Woei Yang.

**Funding acquisition:** Lung Chang.

**Investigation:** Lung Chang.

**Project administration:** Lung Chang.

**Resources:** Tung-Ying Chen and Lung Chang.

**Supervision:** Dar-Shong Lin and Lung Chang.

**Validation:** Tung-Ying Chen, Horng-Woei Yang, and Zo-Darr Huang.

**Visualization:** Tung-Ying Chen, and Horng-Woei Yang.

**Writing – original draft:** Tung-Ying Chen, Horng-Woei Yang, and Lung Chang.

**Writing – review & editing:** Tung-Ying Chen, Horng-Woei Yang, Zo-Darr Huang, Dar-Shong Lin, and Lung Chang.

**Conceptualization:** Horng-Woei Yang, Lung Chang, Tung-Ying Chen.

**Data curation:** Horng-Woei Yang, Lung Chang, Tung-Ying Chen, Zo-Darr Huang.

**Formal analysis:** Horng-Woei Yang, Tung-Ying Chen.

**Funding acquisition:** Lung Chang.

**Investigation:** Lung Chang.

**Project administration:** Lung Chang.

**Resources:** Lung Chang, Tung-Ying Chen.

**Supervision:** Dar-Shong Lin, Lung Chang.

**Validation:** Horng-Woei Yang, Tung-Ying Chen, Zo-Darr Huang.

**Visualization:** Horng-Woei Yang, Tung-Ying Chen.

**Writing – original draft:** Horng-Woei Yang, Lung Chang, Tung-Ying Chen.

**Writing – review & editing:** Dar-Shong Lin, Horng-Woei Yang, Lung Chang, Tung-Ying Chen, Zo-Darr Huang.

## Supplementary Material


