# Supplementary material for: HIV fragments detected in Kaposi sarcoma tumor cells in HIV-infected patients
Source: Medicine (Baltimore). 2022 Oct 28;101(43):e31310. doi: 10.1097/MD.0000000000031310 (PMC9622637; doi:10.1097/MD.0000000000031310)
Supplement: Supplementary file 1 [file medi-101-e31310-s001.pdf]

Supplementary Table 2

Applied ddPCR primer and probe sets.

Oligonucleotide ID	DNA sequence
HIV Gag[18]	
HIV SCA 6F	5’-CAT GTT TTC AGC ATT ATC AGA AGG A-3’
HIV SCA 84R	5’-TGC TTG ATG TCC CCC CAC T-3’
HIV SCA probe 32Hex	5’-/56-HEX/-CCA CCC CAC AAG ATT TAA ACA CCA TGC TAA-/ZEN//3IaBkFQ/-3’
HIV Pol	
HIV Pol299F	5’-GCA CTT TAA ATT TTC CCA TTA GTC CTA-3’
HIV Pol348R	5’-CAA ATT TCT ACT AAT GCT TTT ATT TTT TC-3’
HIV Pol probe	5’-/56-FAM/-AAG CCA GGA ATG GAT GGC C-/ZEN//3IaBkFQ/-3’
