# Supplementary material for: HIV fragments detected in Kaposi sarcoma tumor cells in HIV-infected patients
Source: Medicine (Baltimore). 2022 Oct 28;101(43):e31310. doi: 10.1097/MD.0000000000031310 (PMC9622637; doi:10.1097/MD.0000000000031310)
Supplement: Supplementary file 2 [file medi-101-e31310-s002.pdf]

Supplementary Table 1

Counting of positive signal from ddPCR (Pol, Gag) and RNAscope under high-power field.

Sample	Pol	Gag	HIV (10hpf)	KSHV (10hpf)
1	0	0	2	1
2	32	0	1	17
3	0	0	10	43
4	0	0	1	1
5	0	0	1	45
6	0	0	1	1
7	16	30	0	0
8	0	24	2	37
9	32	0	3	0
10	0	0	1	3
11	11.8	0	1	0
12	38	0	5	35
13	0	0	1	0
14	0	0	14	63
15	11	26	3	2
16	0	0	2	0
17	22	0	2	0
18	16	0	3	19
19	0	0	21	>100
20	0	10.4	8	>100
21	40	24	4	>100
22	6.2	0	5	0

23	4	0	1	4
24	12.4	0	3	39
C1	0	0	0	>100
C2	0	0	0	>100
C3	0	0	0	>100
pLP1-10	26	38		

hpf: high-power field

Supplementary Table 2

Applied ddPCR primer and probe sets.

Oligonucleotide ID	DNA sequence
HIV Gag[18]	
HIV SCA 6F	5’-CAT GTT TTC AGC ATT ATC AGA AGG A-3’
HIV SCA 84R	5’-TGC TTG ATG TCC CCC CAC T-3’
HIV SCA probe 32Hex	5’-/56-HEX/-CCA CCC CAC AAG ATT TAA ACA CCA TGC TAA-/ZEN//3IaBkFQ/-3’
HIV Pol	
HIV Pol299F	5’-GCA CTT TAA ATT TTC CCA TTA GTC CTA-3’
HIV Pol348R	5’-CAA ATT TCT ACT AAT GCT TTT ATT TTT TC-3’
HIV Pol probe	5’-/56-FAM/-AAG CCA GGA ATG GAT GGC C-/ZEN//3IaBkFQ/-3’
